# Concurrent use of statins decreases major bleeding and intracerebral hemorrhage in non-valvular atrial fibrillation patients taking direct oral anticoagulants—A nationwide cohort study

**DOI:** 10.3389/fcvm.2022.969259

**Published:** 2022-08-08

**Authors:** Hsin-Hsu Wu, Shang-Hung Chang, Tsong-Hai Lee, Hui-Tzu Tu, Chi-Hung Liu, Ting-Yu Chang

**Affiliations:** ^1^Department of Nephrology, Kidney Research Center, Chang Gung Memorial Hospital, Taoyuan, Taiwan; ^2^College of Medicine, Chang Gung University, Taoyuan, Taiwan; ^3^Center for Big Data Analytics and Statistics, Chang Gung Memorial Hospital, Taoyuan, Taiwan; ^4^Cardiovascular Department, Chang Gung Memorial Hospital, Taoyuan, Taiwan; ^5^Department of Neurology, Stroke Section, Chang Gung Memorial Hospital, Taoyuan, Taiwan

**Keywords:** atrial fibrillation, DOACs, direct-acting oral anticoagulant, major bleeding, intracerebral hemorrhage, statin

## Abstract

**Background:**

Statins are frequently prescribed with direct oral anticoagulants (DOACs), and previous studies have raised concerns about the increased risk of intracerebral hemorrhage or other major bleeding in concurrent statins and DOACs use. The objective of this study is to evaluate the risk of major bleeding in non-valvular atrial fibrillation patients taking DOACs with or without statins.

**Methods:**

This nationwide, retrospective cohort study used data from the Taiwan National Health Insurance Research Database, enrolled a total of 90,731 non-valvular atrial fibrillation patients receiving rivaroxaban, dabigatran, apixaban or edoxaban from January 1st, 2012 to December 31st, 2017. Major bleeding was defined as a hospitalization or emergency department visit with a primary diagnosis of intracerebral hemorrhage, gastrointestinal tract bleeding, urogenital tract bleeding, or other sites of bleeding. Adjusted incidence rate ratios (IRR) and differences of major bleeding between person-quarters of DOACs with or without statins were estimated using a Poisson regression and inverse probability of treatment weighting using the propensity score.

**Results:**

50,854 (56.0%) of them were male with a mean age of 74.9 (SD, 10.4) years. Using DOACs without statins as a reference, the adjusted IRR for all major bleedings in concurrent use of DOACs and statins was 0.8 (95% CI 0.72–0.81). Lower major bleeding risk was seen in both low-to-moderate-intensity statins (IRR: 0.8, 95% CI 0.74–0.84) and high-intensity statins (IRR: 0.8, 95% CI 0.74–0.88). Concurrent use of DOACs and statins decreased the risk for intracerebral hemorrhage with an IRR of 0.8 (95% CI 0.66–0.93), and gastrointestinal tract bleeding with an IRR of 0.7 (95% CI 0.69–0.79). The protective effect of statins on intracerebral hemorrhage was observed only in female patients (IRR 0.67, 95% CI 0.51–0.89), but not in male patients (IRR 0.87, 95% CI 0.70–1.08).

**Conclusions:**

Among non-valvular atrial fibrillation patients who were taking DOACs, concurrent use of statins decreased major bleeding risk, including intracerebral hemorrhage and gastrointestinal tract bleeding. Considering this and other cardioprotective effects, statins should be considered in all eligible patients prescribed with DOACs.

## Introduction

Atrial fibrillation (AF) is the most common arrhythmia; patients having AF and other comorbidities, especially those with a high CHA_2_DS_2_-VASc score, are at an increased risk of embolic stroke (1). Oral anticoagulation is a widely-used management strategy for AF, and direct oral anticoagulants (DOACs) are often prescribed due to their favorable efficacy and safety profiles compared to vitamin K antagonists (VKA) (2–5). However, their bleeding risks cannot be neglected (6), especially in patients with multiple medications and potential drug-drug interactions. Hyperlipidemia is another important risk factor for stroke and cardiovascular disease. Intensive lipid control with statins is widely recommended for patients at high risks (7, 8). The relationship between statins and bleeding risk, especially intracerebral hemorrhage (ICH), has been debated for years with inconsistent findings. In the SPARCL trial, high-dose statins were associated with a slightly increased ICH incidence (9). A meta-analysis of several randomized controlled trials also showed that statins might increase the risk of ICH in patients with a history of stroke or cardiovascular disease (10). However, in other meta-analyses and cohort studies, statins were not associated with a higher ICH risk (11–13). Even in patients with a history of ICH, statins may not increase the risk of recurrent ICH (14). Expert consensus currently supports the notion that the benefits of statins in preventing cardiovascular events outweighs the possible increased risk of ICH (15).

AF and dyslipidemia are common co-morbidities and thus DOACs and statins are frequently prescribed together. An earlier study reported reduced ICH risk in patients taking atorvastatin and DOACs (16), while other research found that dabigatran users had a higher risk of major hemorrhage when prescribed with simvastatin or lovastatin than with other statins (17). Whether concurrent use of different intensities of statins and DOACs increases the risk of ICH or other major bleeding remains unclear. To clarify this, we used a nationwide cohort of non-valvular AF patients to estimate the bleeding risk of simultaneous DOACs and different intensities of statins use.

## Methods

### Study design

#### Ethics approval and consent to participate

This study was approved by the institutional review board of the Chang Gung Memorial Hospital, Taoyuan, Taiwan (IRB No.: 201701397B1). Informed consent was waived as no identifiable information was used in this study.

#### Data source

This retrospective cohort study investigated all patients that were registered in the Taiwan National Health Insurance (NHI) program. The Taiwan NHI program was founded in 1995 as a single-payer insurance system and is mandatory for all citizens and foreigners living in Taiwan for more than 6 months. By the end of 2017, ~23 million beneficiaries were registered in the NHI with a coverage rate of more than 99.5%. The NHI maintains an anonymous database, National Health Insurance Research Database (NHIRD), since 1995 that records comprehensive characteristics of all beneficiaries, including medical diagnoses, detailed prescriptions, examinations, surgical procedures, and incurred fees. From 1997 to 2015, the *International Classification of Diseases, 9th Revision, Clinical Modification (ICD-9-CM)* codes were used for diagnosis and procedure coding, and the *International Classification of Diseases, 10th Revision, Clinical Modification (ICD-10-CM)* codes have been used since 2016.

#### Data availability

The data repository of NHIRD is Health and Welfare Data Science Center, Taiwan. Researchers can obtain the data through formal application to the Health and Welfare Data Science Center, Department of Statistics, Ministry of Health and Welfare, Taiwan (https://dep.mohw.gov.tw/DOS/np-2500-113.html).

#### Study population

**Figure 1:**
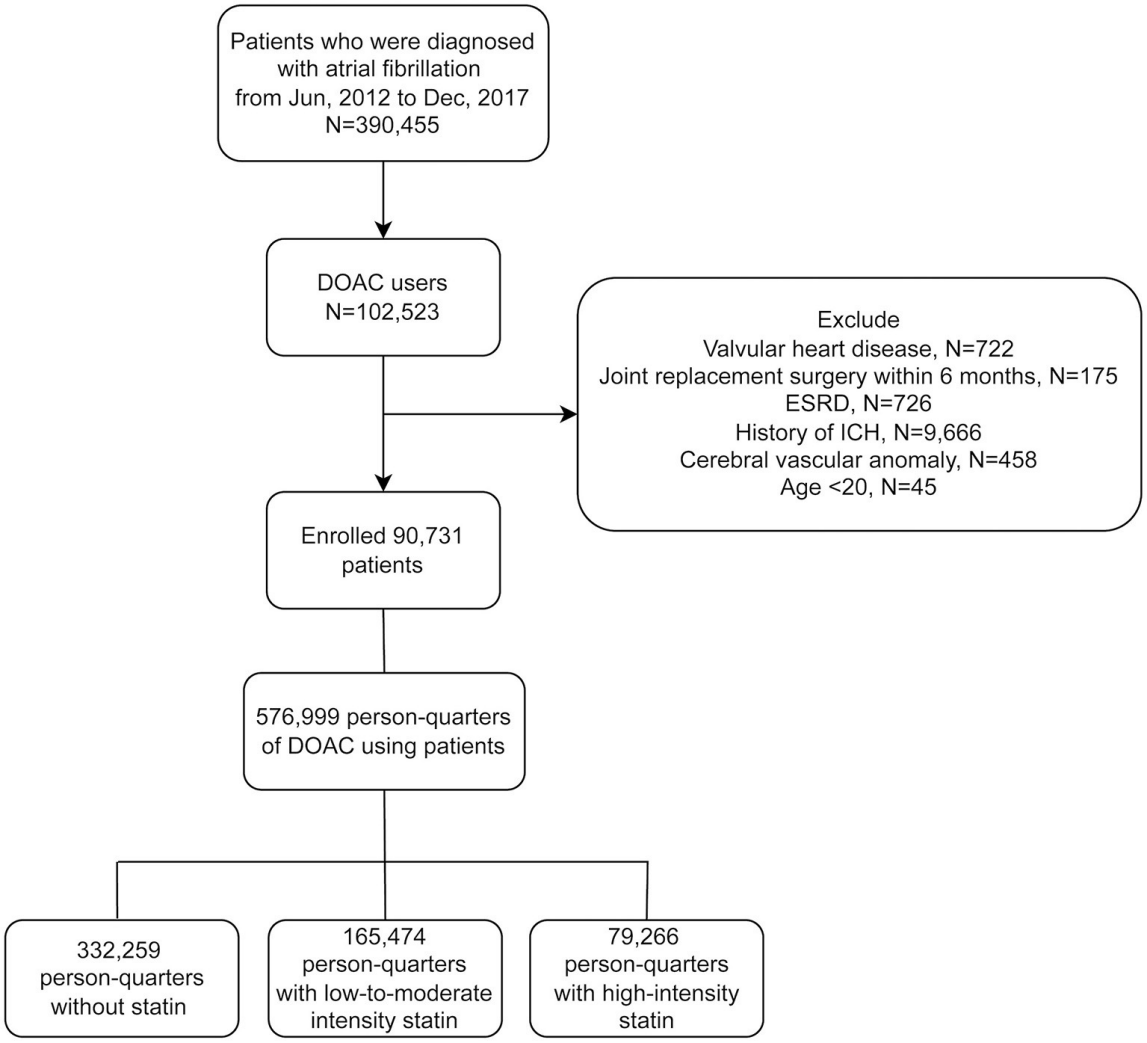
Flowchart of the study protocol. DOAC, direct oral anticoagulant; ESRD, end-stage renal disease; ICH, intracerebral hemorrhage.

All patients with 2 consecutive records of non-valvular AF diagnosis (*ICD-9-CM* code 427.31 or *ICD-10-CM* code I48) (16) and at least one DOAC prescription (rivaroxaban, dabigatran, apixaban or edoxaban) from January 1st 2012 to December 31st 2017 were enrolled. Patients who had valvular heart disease, joint surgery within 6 months prior to the first DOAC prescription, end-stage renal disease, previous ICH history, or cerebral vascular anomaly were excluded (Figure 1).

#### Person-quarters and statins using

We partitioned each calendar year into four quarters for each patient and each year after the first prescription of DOAC. One person-quarter (PQ) was used as an analytical unit. We used PQs because medications for chronic illnesses were refilled with a maximum duration of 3 months per the Taiwan NHI reimbursement policy (16, 18). Medications and covariates were assessed for each PQ, and PQs exposed to DOACs with or without statins were identified. The major bleeding risks of PQs exposed to statins and DOACs were compared with PQs exposed to DOACs alone. PQ with statin using were further subdivided into low-to-moderate- and high-intensity statin groups. High-intensity statins were defined as atorvastatin 40–80 mg or rosuvastatin 20 mg per day (Supplementary Table 1) (19). This design may help to overcome the complexity of prescription adjustments (changing the intensities of statins or stopping statins due to side effects) in real-world practice and to identify the events under specific medications.

#### Outcomes

The primary outcome was major bleeding, defined as a hospitalization or an emergency department visit with a primary diagnosis of ICH, gastrointestinal tract (GI) bleeding, urogenital bleeding, or other sites of bleeding (listed in Supplementary Table 1). Within the PQ, the time-points of DOACs prescription, statin prescription, and bleedings were all recorded, as to ensure that only bleedings after DOACs prescription were counted, and only bleedings after statin prescription were recorded as bleeding events with statin using. Examples are shown in Supplementary Figure.

#### Covariates

Patient demographics, including sex, age, socioeconomic status, occupation, comorbidities, and relevant medications were identified as covariates. Comorbidities were the components of the Charlson comorbidity index (myocardial infarction, congestive failure, peripheral vascular disease, stroke, transient ischemic attack, dementia, chronic obstructive lung disease, connective tissue disease, peptic ulcer disease, liver disease, diabetes mellitus, moderate to severe chronic kidney disease, cancer, leukemia, lymphoma, acquired immune-deficient syndrome). We also included medications of proton pump inhibitors, warfarin, non-steroidal anti-inflammatory drugs, glucocorticoids, aspirin, clopidogrel, ticlopidine and HAS-BLED score as covariates. In our study, HAS-BLED score was ranged from 0 to 8 and calculated by assigning one point each for hypertension, chronic kidney disease, mild to severe liver disease, stroke history, age > 65 years, antiplatelet use, and non-steroidal anti-inflammation drug use (Supplementary Table 2). Since the international normalized ratio (INR) was not available in the NHIRD, we did not include labile INR for HAS-BLED scoring, consistent with other registry database studies (20–22). These covariates were assessed for each PQ on the first date of the pertinent PQ and are listed in Supplementary Table 3.

#### Propensity score

The selection of one medication over another is confounded by the indication and may result in a non-random treatment allocation. We used the inverse probability of treatment weighting by the propensity score to account for this bias (23). The propensity score was the probability that a patient was prescribed the concurrent medication during a PQ. For each PQ, a specific propensity score was calculated according to the aforementioned covariates that were pertinent to the first date of the PQ.

### Statistical analysis

We used generalized estimating equations (24) for a Poisson regression model to account for intra-individual correlation across PQs to calculate the incidence rate, incidence rate ratios (IRR), incidence rate difference (IRD) and 95% confidence intervals (CIs) that considered the inverse probability of treatment weighting using the propensity score.

Data from patients without a valid insurance status were considered missing data and were excluded from this study (estimated at <0.1%). The analysis was performed using SAS (SAS Institute) version 9.4.

## Results

From 2012 to 2017, a total of 390,455 AF patients were identified. Of these, 102,523 were DOACs users. Only adult patients (age ≥ 20) were enrolled. Patients receiving joint replacement surgery or prosthetic valve surgery within 6 months before the first DOACs prescription (*N* = 175) were excluded, as well as those with valvular heart disease (*N* = 722), because the focus of this study was non-valvular AF. Patients with end-stage renal disease (ESRD) (*N* = 726) were excluded due to excessive bleeding risks. Finally, we excluded patients who had previous intracerebral hemorrhage (ICH) (*N* = 9,666) or cerebral vascular anomaly (*N* = 458), because these patients may carry excessive intracranial bleeding risks (25). A total of 90,731 patients were enrolled, and a total of 576,999 PQs were identified. There was a total of 165,474 PQs for low-to-moderate-intensity statin group and 79,266 PQs for high-intensity statin group (Figure 1).

### Demographics

Table 1 shows the demographic data of our study population. The mean age was 74.94 years (SD, 10.39) and 56.05% of them were male. The HAS-BLED score was 3.00 (SD, 1.13). The average CHA_2_DS_2_-VASc score [congestive heart failure, hypertension, age ≥75 years (two points), age 65–74 years, diabetes mellitus, prior stroke or transient ischemic attack or thromboembolism (two points), vascular disease history, female] was 4.59 (SD, 1.73). Half of our patients had congestive heart failure (51.71%), and more than one third of them had cerebral vascular disease (46.07%), or diabetes mellitus (41.28%).

**Table 1:** Characteristics, comorbidities and medications among patients with non-valvular atrial fibrillation using DOACs.

**DOAC users (*****n*** = **90,731)**		
**Characteristics**	**Mean**	**SD or (%)**
Age	74.94	±10.39
CHA_2_DS_2_-VASc	4.59	±1.73
HAS-BLED	3.00	±1.13
Charlson comorbidity index	4.37	±2.34
**Gender**		
Female	39,877	(43.95%)
Male	50,854	(56.05%)
**Occupation**		
Dependents of the insured individuals	27,355	(30.15%)
Civil servants, teachers, military personnel and veterans	2,032	(2.24%)
Non-manual workers and professionals	9,210	(10.15%)
Manual workers	31,393	(34.60%)
Other	20,741	(22.86%)
**Residence**		
Urban	47,878	(52.77%)
Suburban	29,622	(32.65%)
Rural	12,605	(13.89%)
Unknown	626	(0.69%)
**Income**		
Quintile 1	26,482	(29.19%)
Quintile 2	1,326	(1.46%)
Quintile 3	37,663	(41.51%)
Quintile 4	8,159	(8.99%)
Quintile 5	16,905	(18.63%)
Unknown	196	(0.22%)
**Comorbidities**		
Hypertension	78,973	(87.04%)
Myocardial infarction	5,773	(6.36%)
Congestive heart failure	46,914	(51.71%)
Peripheral vascular disease	8,880	(9.79%)
Coronary revascularization	1,574	(1.73%)
Cerebrovascular disease	41,803	(46.07%)
Ischemic stroke	31,286	(34.48%)
Transient ischemic attack	11,329	(12.49%)
Hemiplegia or paraplegia	3,731	(4.11%)
Dementia	9,730	(10.72%)
Diabetes mellitus	37,453	(41.28%)
Diabetes with complications	13,231	(14.58%)
Chronic pulmonary disease	46,791	(51.57%)
Chronic obstructive pulmonary disease	42,203	(46.51%)
Anemia	13,810	(15.22%)
Rheumatic disease	6,945	(7.65%)
Chronic kidney disease	23,686	(26.11%)
Renal disease	17,278	(19.04%)
Peripheral arterial occlusive disease	2,386	(2.63%)
Peptic ulcer disease	50,457	(55.61%)
Mild liver disease	31,947	(35.21%)
Moderate or severe liver disease	282	(0.31%)
Cancer	12,889	(14.21%)
Metastatic solid tumor	1,267	(1.40%)
Human immunodeficiency virus infection	22	(0.02%)
Percutaneous coronary intervention	7,871	(8.68%)
Coronary artery bypass surgery	968	(1.07%)
**Medication**		
Aspirin	40,985	(45.17%)
Clopidogrel	10,646	(11.73%)
NSAIDs	21,670	(23.88%)
PPI	8,930	(9.84%)
Ticlopidine	2,281	(2.51%)
Warfarin	23,718	(26.14%)

CHA_2_DS_2_-VASc, Congestive heart failure, Hypertension, Age ≥75 (2 points), Diabetes Mellitus, Prior Stroke or Transient Ischemic Attack (2 points), Vascular Disease, Female; HAS-BLED, hypertension, chronic kidney disease, mild liver disease or moderate or severe liver disease, stroke, history of bleeding, Age >65, antiplatelet or non-steroidal anti-inflammation drug use; NSAID, non-steroidal anti-inflammation drug; PPI, proton pump inhibitor.

### All major bleeding

Major bleeding was defined as bleeding requiring hospitalization or an emergency department visit. During follow-up, 4,580 major bleeding events occurred among 332,259 no-statin PQs, while 2,289 major bleeding events were recorded among 238,923 statin-using PQs. The adjusted incidence rate for all major bleeding events of no-statin PQs was 50.86 (95% CI 48.73–53.09) per 1,000 person-year and 38.71 (95% CI 37.02–40.48) per 1,000 person-year for statin-using. Lower bleeding incidence was observed in both high-intensity and low-to-moderate-intensity statin-using PQs (incidence rate of 40.62 and 38.30 per 1,000 person-year, respectively) than in no-statin PQs. Compared with PQs of DOACs alone, the adjusted IRR of major bleeding for a DOACs with statins, high-intensity statins, and low-to-moderate-intensity statins were 0.76 (95% CI 0.72–0.81), 0.81 (95% CI 0.74–0.88), and 0.75 (95% CI 0.70–0.80), respectively. The adjusted IRD per 1,000 person-year of major bleeding among concurrent statin and DOACs use was −12.15 (95% CI −14.94 to −9.37) (Table 2).

**Table 2:** Major bleeding among DOAC using patients.

**Major bleeding**							
	**Concurrent statin**	**No. of PQs**	**No. of events**	**Crude incidence rate (per 1,000 person-year)** **(95% CI)**	**Adjusted incidence rate** **(per 1,000 person-year)** **(95% CI)**	**Adjusted IRR** **(95% CI)**	**Adjusted IRD (per 1,000 person-year)** **(95% CI)**
All	No statin	332259	4,580	55.69 (53.95–57.48)	50.86 (48.73–53.09)	1	
	All intensity	238923	2,289	38.73 (36.03–40.49)	38.71 (37.02–40.48)	0.76 (0.72–0.81)	−12.15 (−14.94 to −9.37)
	High-intensity	79266	796	40.67 (37.70–43.87)	40.62 (37.65–43.82)	0.81 (0.74–0.88)	−9.52 (−13.32 to −5.72)
	Low-to-moderate-intensity	165474	1,568	38.32 (36.33–40.43)	38.30 (36.31–40.40)	0.75 (0.70–0.80)	−12.74 (−15.71 to −9.78)
Male	No statin	190084	2,444	51.89 (49.70–54.19)	47.39 (44.69–50.25)	1	
	All intensity	135304	1,260	37.61 (35.41–39.95)	37.58 (35.38–39.92)	0.79 (0.73–0.86)	−9.81 (−13.40 to −6.22)
	High-intensity	45017	434	39.00 (35.21–43.19)	38.93 (35.15–43.11)	0.83 (0.74–0.94)	−7.95 (−12.85 to −3.05)
	Low-to-moderate-intensity	93569	868	37.49 (34.87–40.31)	37.45 (34.83–40.26)	0.79 (0.72–0.87)	−9.96 (−13.80 to −6.12)
Female	No statin	141669	2,133	60.89 (58.12–63.80)	57.39 (53.95–61.06)	1	
	All intensity	103070	1,023	40.16 (37.57–42.93)	40.16 (37.57–42.93)	0.70 (0.64–0.77)	−17.24 (−21.68 to −12.79)
	High-intensity	34017	362	43.17 (38.54–48.34)	43.13 (38.51–48.30)	0.76 (0.67–0.87)	−13.52 (−19.56 to −7.48)
	Low-to-moderate-intensity	71558	694	39.27 (36.27–42.51)	39.26 (36.26–42.50)	0.68 (0.62–0.75)	−18.26 (−22.95 to −13.56)

*DOAC, direct oral anticoagulant; PQ, person-quarter; CI, confident interval; IRR, incidence rate ratio; IRD, incidence rate difference*.

**Figure 2:**
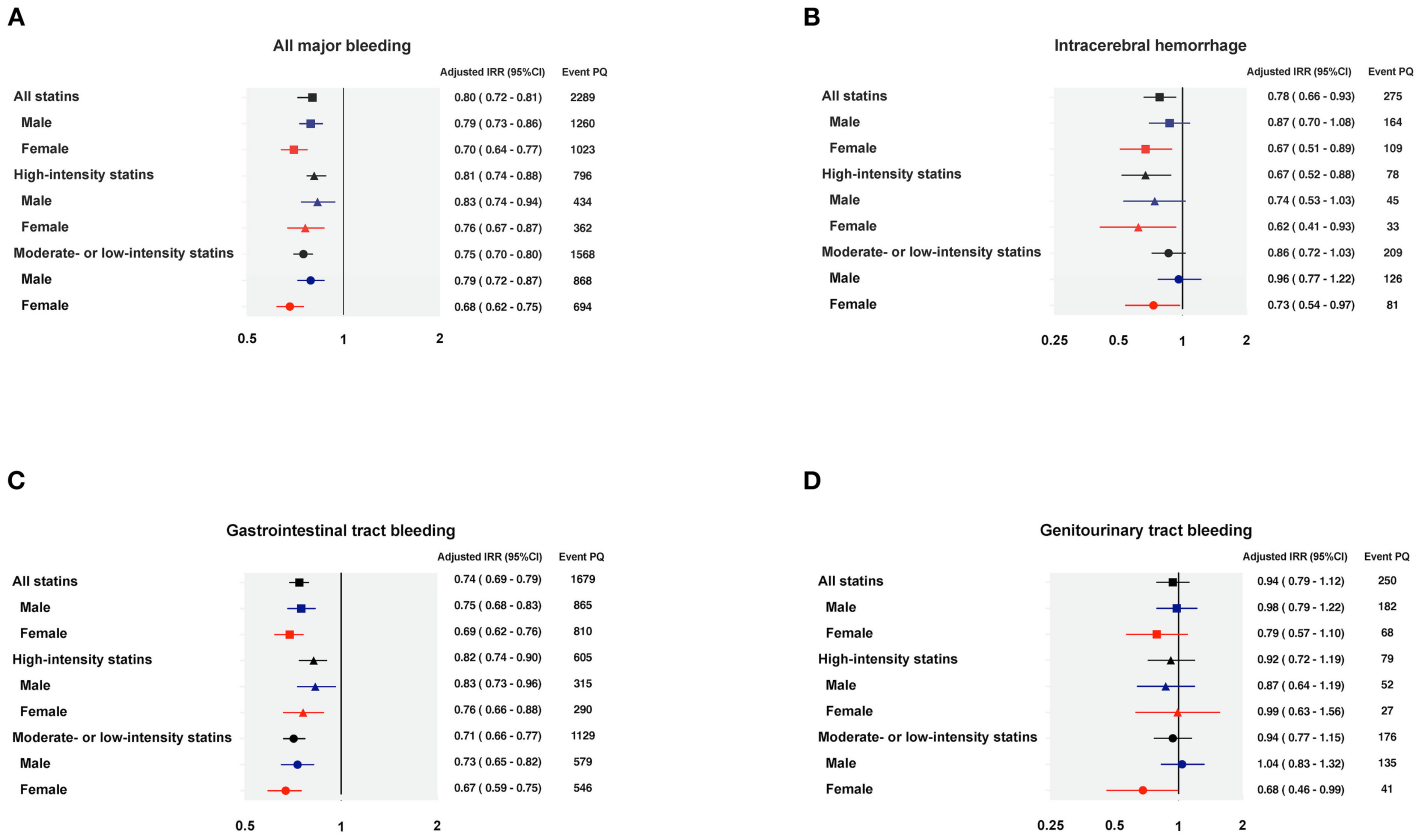
Forest plots of the adjusted incidence rate ratio regarding major bleedings. **(A)** All major bleeding; **(B)** Intracerebral hemorrhage; **(C)** Gastrointestinal tract bleeding; **(D)** Genitourinary tract bleeding. IRR, incidence rate ratio; PQ, person-quarter.

Examining sex differences, the IRR for all major bleeding in statin-using males was 0.79 (95% CI 0.73–0.86), and 0.70 (95% CI 0.64–0.77) in statin-using females. The IRD for all major bleeding in statin-using males was −9.81 (95% CI −13.40 to −6.22) per 1,000 person-year, and −17.24 (95% CI −21.68 to −12.79) per 1,000 person-year in statin-using females. Figure 2 demonstrated the forest plots of major bleeding risks among different sites.

### Intracerebral hemorrhage

The adjusted incidence rate for ICH was 5.95 (95% CI 5.26–6.73) per 1,000 person-year among no-statin PQs, and 4.65 (95% CI 4.12–5.25) per 1,000 person-year among statin-using PQs (Table 3). A lower ICH risk was observed in concurrent statin and DOACs use, especially in high-intensity statin group. The IRR of concurrent high-intensity statin use was 0.67 (95% CI 0.52–0.88) and the IRD was−1.93 (95% CI −3.13 to −0.73). The IRR of concurrent low-to-moderate-intensity statin use was 0.86 (95% CI 0.72–1.03) and the IRD was −0.84 (95% CI −1.84 to 0.17) per 1,000 person-year. While statin-using did not show significant difference on ICH risk among male DOAC users [IRR 0.87 (95% CI 0.70–1.08); IRD −0.72 (95% CI −1.86 to 0.41)] per 1,000 person-year, combined prescription of statin decreased the risk of ICH in female DOAC users with an IRR of 0.67 (95% CI 0.51–0.89) and IRD of −2.07 (95% CI −3.56 to −0.59) per 1,000 person-year. It is of note that high-intensity statins seemed to provide better protection among female patients, with an IRR of 0.62 (95% CI 0.41–0.93) and IRD of −2.44 (95% CI −4.35 to −0.54) per 1,000 person-year (Table 3).

**Table 3:** Intracerebral hemorrhage among DOAC using patients.

**Intracerebral hemorrhage**							
	**Concurrent statin**	**No. of PQs**	**No. of events**	**Crude incidence rate (per 1,000 person-year) (95% CI)**	**Adjusted incidence rate (per 1,000 person-year) (95% CI)**	**Adjusted IRR (95% CI)**	**Adjusted IRD (per 1,000 person-year) (95% CI)**
All	No statin	332259	492	5.98 (5.46–6.55)	5.95 (5.26–6.73)	1	
	All intensity	238923	275	4.65 (4.12–5.25)	4.65 (4.12–5.25)	0.78 (0.66–0.93)	−1.30 (−2.22 to −0.38)
	High-intensity	79266	78	3.98 (3.17–4.99)	3.98 (3.17–5.00)	0.67 (0.52–0.88)	−1.93 (−3.13 to −0.73)
	Low-to-moderate-intensity	165474	209	5.11 (4.44–5.87)	5.1 (4.44–5.86)	0.86 (0.72–1.03)	−0.84 (−1.84 to 0.17)
Male	No statin	190084	282	5.99 (5.31–6.75)	5.62 (4.84–6.52)	1	
	All intensity	135304	164	4.91 (4.20–5.74)	4.9 (4.19–5.73)	0.87 (0.70–1.08)	−0.72 (−1.86 to 0.41)
	High-intensity	45017	45	4.05 (3.01–5.46)	4.04 (3.00–5.45)	0.74 (0.53–1.03)	−1.45 (−2.92 to 0.01)
	Low-to-moderate-intensity	93569	126	5.45 (4.56–6.52)	5.44 (4.55–6.51)	0.96 (0.77–1.22)	−0.2 (−1.48 to 1.08)
Female	No statin	141669	210	5.99 (5.21–6.88)	6.34 (5.22–7.71)	1	
	All intensity	103070	109	4.27 (3.52–5.19)	4.27 (3.52–5.18)	0.67 (0.51–0.89)	−2.07 (−3.56 to −0.59)
	High-intensity	34017	33	3.91 (2.76–5.56)	3.92 (2.76–5.57)	0.62 (0.41–0.93)	−2.44 (−4.35 to −0.54)
	Low-to-moderate-intensity	71558	81	4.57 (3.65–5.72)	4.57 (3.65–5.72)	0.73 (0.54–0.97)	−1.73 (−3.30 to −0.16)

*DOAC, direct oral anticoagulant; PQ, person-quarter; CI, confident interval; IRR, incidence rate ratio; IRD, incidence rate difference*.

### Gastrointestinal tract bleeding

Concurrent statin use reduced the risk of gastrointestinal (GI) tract bleeding in patients taking DOACs, regardless of statin intensities or sex differences. Compared to no-statin PQs, the IRR during statin-using PQs was 0.74 (95% CI 0.69–0.79) and the IRD was −10.12 (95% CI −12.51 to −7.73) per 1,000 person-year (Table 4). The IRRs were 0.82 (95% CI 0.74–0.90) and 0.71 (95% CI 0.66–0.77) under the prescription of high-intensity statin and low-to-moderate-intensity statin, respectively. There were no sex-differences regarding the protective effect of concurrent statin use in GI tract bleeding (Table 4).

**Table 4:** Gastrointestinal tract bleeding among DOAC using patients.

**Gastrointestinal tract bleeding**							
	**Concurrent statin**	**No. of PQs**	**No. of events**	**Crude incidence rate (per 1,000 person-year) (95% CI)**	**Adjusted incidence rate (per 1,000 person-year) (95% CI)**	**Adjusted IRR (95% CI)**	**Adjusted IRD (per 1,000 person-year) (95% CI)**
All	No statin	332259	3533	43 (41.43–44.56)	38.5 (36.68–40.42)	1	
	All intensity	238923	1679	28.4 (26.95–29.94)	28.4 (26.93–29.92)	0.74 (0.69–0.79)	−10.12 (−12.51 to −7.73)
	High-intensity	79266	605	30.9 (28.34–33.77)	30.9 (28.30–33.72)	0.82 (0.74–0.90)	−6.97 (−10.25 to −3.69)
	Low-to-moderate-intensity	165474	1129	27.6 (25.89–29.39)	27.6 (25.87–29.36)	0.71 (0.66–0.77)	−11.13 (−13.68 to −8.58)
Male	No statin	190084	1796	38.13 (36.23–40.12)	34.13 (31.89–36.52)	1	
	All intensity	135304	865	25.8 (23.97–27.76)	25.76 (23.94–27.72)	0.75 (0.68–0.83)	−8.37 (−11.35 to −5.39)
	High-intensity	45017	315	28.31 (25.10–31.94)	28.25 (25.04–31.87)	0.83 (0.73–0.96)	−5.61 (−9.74 to −1.47)
	Low-to-moderate-intensity	93569	579	24.97 (22.85–27.30)	24.93 (22.80–27.25)	0.73 (0.65–0.82)	−9.17 (−12.35. −6.00)
Female	No statin	141669	1735	49.56 (47.04–52.22)	46.44 (43.36–49.74)	1	
	All intensity	103070	810	31.82 (29.51–34.32)	31.83 (29.51–34.33)	0.69 (0.62–0.76)	−14.46 (−18.47 to −10.45)
	High-intensity	34017	290	34.61 (30.46–39.32)	34.59 (30.44–39.30)	0.76 (0.66–0.88)	−11.01 (−16.44 to −5.57)
	Low-to-moderate-intensity	71558	546	30.91 (28.24–33.84)	30.91 (28.24–33.83)	0.67 (0.59–0.75)	−15.53 (−19.77 to −11.29)

DOAC, direct oral anticoagulant; PQ, person-quarter; CI, confident interval; IRR, incidence rate ratio; IRD, incidence rate difference.

### Urogenital tract and other sites bleeding

DOACs use alone and co-medication with statins had similar incidence rates of urogenital tract bleeding events. Compared with PQs of no-statin use, the IRR for urogenital tract bleeding during statin-using PQs was 0.94 (95% CI 0.79–1.12). No significant sex difference was observed in the incidence of urogenital tract bleeding. For statin-using males, the IRR was 0.98 (95% CI 0.79–1.22) for urogenital tract bleeding, while for statin-using females, the IRR was 0.79 (95% CI 0.57–1.10) (Table 5).

**Table 5:** Genitourinary tract bleeding among DOAC using patients.

**Urogenital tract bleeding**							
	**Concurrent statin**	**No. of PQs**	**No. of events**	**Crude incidence rate (per 1,000 person-year) (95% CI)**	**Adjusted incidence rate (per 1,000 person-year) (95% CI)**	**Adjusted IRR (95% CI)**	**Adjusted IRD (per 1,000 person-year) (95% CI)**
All	No statin	332259	425	5.12 (4.64–5.66)	4.46 (3.94–5.05)	1	
	All intensity	238923	250	4.19 (3.68–4.77)	4.19 (3.68–4.77)	0.94 (0.79–1.12)	−0.27 ( −1.04 to 0.50)
	High-intensity	79266	79	4.00 (3.20–4.99)	3.99 (3.20–4.99)	0.92 (0.72 −1.19)	−0.32 (−1.37 to 0.72)
	Low-to-moderate-intensity	165474	176	4.26 (3.64–4.97)	4.26 (3.64–4.97)	0.94 (0.77 −1.15)	−0.26 (−1.12 to 0.61)
Male	No statin	190084	288	6.07 (5.38–6.85)	5.48 (4.72–6.37)	1	
	All intensity	135304	182	5.39 (4.63–6.28)	5.39 (4.63–6.28)	0.98 (0.79 −1.22)	−0.09 (−1.25 to 1.07)
	High-intensity	45017	52	4.63 (3.53–6.07)	4.63 (3.53–6.07)	0.87 (0.64 −1.19)	−0.68 (−2.17 to 0.82)
	Low-to-moderate-intensity	93569	135	5.78 (4.83–6.92)	5.78 (4.83–6.92)	1.04 (0.83 −1.32)	0.24 (−1.09 to 1.56)
Female	No statin	141669	137	3.87 (3.24–4.62)	3.37 (2.69–4.22)	1	
	All intensity	103070	68	2.64 (2.07–3.35)	2.64 (2.07–3.35)	0.79 (0.57 −1.10)	−0.7 (−1.69 to 0.29)
	High-intensity	34017	27	3.18 (2.16–4.70)	3.18 (2.15–4.69)	0.99 (0.63 −1.56)	−0.02 (−1.46 to 1.42)
	Low-to-moderate-intensity	71558	41	2.29 (1.69–3.10)	2.29 (1.69–3.11)	0.68 (0.46 −0.99)	−1.08 (−2.11 to −0.05)

DOAC, direct oral anticoagulant; PQ, person-quarter; CI, confident interval; IRR, incidence rate ratio; IRD, incidence rate difference.

Concurrent statin use did not increase the risk of bleeding at other sites with an IRR of 0.76 (95% CI 0.52–1.11) (Table 6). No significant differences between statin intensities or sex were found.

**Table 6:** Other sites bleeding among DOAC using patients.

**Other sites bleeding**							
	**Concurrent statin**	**No. of PQs**	**No. of events**	**Crude incidence rate (per 1,000 person-year) (95% CI)**	**Adjusted incidence rate (per 1,000 person-year) (95% CI)**	**Adjusted IRR (95% CI)**	**Adjusted IRD (per 1,000 person-year) (95% CI)**
All	No statin	332259	130	1.56 (1.31–1.86)	1.88 (1.38–2.56)	1	
	All intensity	238923	85	1.42 (1.14–1.78)	1.42 (1.14–1.78)	0.76 (0.52–1.11)	−0.45 (−1.12 to 0.21)
	High-intensity	79266	34	1.72 (1.20–2.45)	1.72 (1.20–2.45)	0.85 (0.52–1.41)	−0.3 (−1.24 to 0.64)
	Low-to-moderate-intensity	165474	54	1.31 (0.99–1.72)	1.31 (0.99–1.72)	0.72 (0.48–1.07)	−0.52 (−1.15 to 0.12)
Male	No statin	190084	78	1.64 (1.31–2.06)	2.06 (1.35–3.14)	1	
	All intensity	135304	49	1.45 (1.08–1.94)	1.45 (1.08–1.94)	0.7 (0.42–1.18)	−0.61 (−1.58 to 0.36)
	High-intensity	45017	22	1.95 (1.26–3.02)	1.95 (1.26–3.02)	0.91 (0.47–1.73)	−0.2 (−1.54 to 1.13)
	Low-to-moderate-intensity	93569	28	1.20 (0.82–1.76)	1.20 (0.82–1.76)	0.6 (0.34–1.03)	−0.81 (−1.72 to 0.10)
Female	No statin	141669	51	1.44 (1.09–1.89)	1.39 (0.99–1.95)	1	
	All intensity	103070	36	1.40 (0.99–1.97)	1.40 (0.99–1.97)	0.99 (0.61–1.61)	−0.02 (−0.70 to 0.67)
	High-intensity	34017	12	1.41 (0.77–2.60)	1.41 (0.77–2.60)	0.95 (0.47–1.95)	−0.07 (−1.09 to 0.95)
	Low-to-moderate-intensity	71558	26	1.45 (0.98–2.16)	1.45 (0.98–2.16)	1.04 (0.62–1.76)	0.06 (−0.68 to 0.81)

DOAC, direct oral anticoagulant; PQ, person-quarter; CI, confident interval; IRR, incidence rate ratio; IRD, incidence rate difference.

## Discussion

This nationwide population-based cohort study showed that concurrent use of statins and DOACs did not increase the risk of major bleeding but decreased ICH and GI bleeding. This is the first large cohort study investigating the risk of major bleedings in patients taking different intensities statins with DOACs.

Reviewing the literature, only a few studies evaluated the risk of major bleeding in AF patients receiving statins and long-term anticoagulant therapy. Evidence shows that concurrent use of statins and VKA (warfarin) did not increase the risk of bleeding, but data with simultaneous DOACs and statins use was controversial (26, 27). An earlier study reported a higher ICH risk in patients taking dabigatran and either simvastatin or lovastatin (17). One observational study also suggested an increased bleeding risk with simultaneous use of DOACs and statins (27). On the other hand, a nationwide study from Taiwan found reduced major bleeding events with concurrent use of atorvastatin and DOACs (16). Another prospective study also observed lower bleeding risk with dabigatran and statin use compared to dabigatran alone (28). Like the above two studies, our data further confirmed that decreased major bleeding risks were observed among DOAC users taking statins, regardless of the intensity. This study may provide a more comprehensive insight with several advantages. First, we included four DOACs (rivaroxaban, dabigatran, apixaban or edoxaban); second, we evaluated the bleeding risk of statins with different intensities, though individual statins were not evaluated due to insufficient users and events of certain statins.

Statins have several pleiotropic effects include improvement of endothelial function, increased nitric oxide bioavailability, and anti-inflammation property (29). The pleiotropic effect of statins is considered a class effect. However, different statins have different metabolism, and this may result in having different drug-drug interaction profiles. Atorvastatin and simvastatin are CYP3A4 substrates, but rosuvastatin, fluvastatin, and pitavastatin are less susceptible to cytochrome P450. DOACs are also CYP3A4 substrates and previous studies have showed atorvastatin did not impact the trough activity of DOACs (30, 31). On the other hand, the absorption of DOACs is dependent on intestinal P-glycoproteins (P-gp), as DOACs are P-gp substrates (32–34). P-gp is an ATP-binding cassette drug efflux transporter and can limit the cellular uptake of drugs from the intestinal lumen into epithelial cells, limit substrate uptake from the blood circulation into the brain, and enhance the excretion of substrates from hepatocytes and renal tubules (35, 36). The serum or tissue concentration of a substrate, such as DOACs, may increase in the presence of P-gp inhibitors. Previous *in vitro* models have shown lovastatin, simvastatin and atorvastatin are P-gp inhibitors while fluvastatin and rosuvastatin only interact with P-gp *in vitro* at high concentrations (37, 38). Despite the possibility of increased serum or tissue DOAC concentrations with concurrent statin use, this study did not find an increased risk of major bleeding but found a protective effect of statins in our DOAC-using population. Hence, there might be other mechanisms that reduced the risk of bleeding during co-prescription of DOACs and statins.

A lower ICH risk was observed in concurrent use of statins and DOACs. ICH is one of the most devastating complications during anticoagulation. Most primary ICH is a manifestation of small vessel disease, with long-standing hypertension and cerebral amyloid angiopathy (CAA) being the primary causes. Hypertensive vasculopathy may cause cerebral small vessel lipohyalinosis (also known as fibrinoid necrosis) and endothelium injury, while CAA is characterized by the deposition of amyloid-β peptide in the walls of small cerebral cortical and leptomeningeal vessels (39). Recently, endothelial cell dysfunction and loss of endothelial nitric oxide have been reported to be associated with cerebrovascular amyloid formation (40). Statins have cholesterol-independent vascular pleiotropic effects and may improve and stabilize endothelial function by increasing the bioavailability of nitric oxide, thereby reducing oxidative stress and inhibiting inflammation (41). This might partly explain why statins reduced ICH incidence *via* their pleiotropic effects in our patients. Another important finding is the different ICH risks with different statin intensities and among different sex. Our study showed a reduction in the risk of ICH was mainly observed in high-intensity statin using. Aggressive management of hyperlipidemia with high-intensity statins could be suggested for AF patients on DOAC therapy and should not be withheld due to concerns of ICH. It is worthy of note that statin might have stronger protective effect on the risk of ICH among female patients. Estrogen level was found to correlate with endothelial function and endothelial nitric oxide synthase expression (42). But this still cannot paint the whole picture why statin use reduced female ICH incidence. Further studies are needed to understand the underlying pathophysiology.

We also found concurrent statin use reduced GI bleeding, regardless of intensity. This result supports an earlier study, which suggested statins might have protective effects against GI bleeding in patients with acute coronary syndrome (43). However, other previous studies showed mixed results. In one claims database study, statin users had a higher risk of GI bleeding than other chronic medication users (44). Another retrospective cohort study reported a higher risk of GI bleeding during rosuvastatin combined with warfarin use compared to other statins (45). More studies are needed to clarify the risk of GI bleeding among simultaneous statins and DOACs use.

Statins did not increase major genitourinary tract bleeding in non-valvular AF patients taking DOACs. An earlier study reported that rosuvastatin had a rate of transient microscopic hematuria <1.5% (46), and another study showed the use of statins was associated with a lower risk of uterine myoma and menorrhagia (47). Considering this, statin use would not be expected to further increase genitourinary tract bleeding risk among DOAC users.

### Limitations

Our study has several limitations. First, this was a claims database cohort study and coding inaccuracy might exist, though the coding accuracy of AF and other comorbidities in NHIRD has been validated before (48–50). Second, this study was conducted in Taiwan and more than 95% of the general population was Han-Chinese during the study period. Asians have a high incidence of ICH (51, 52), and thus the ability to generalize these results to other ethnicities is limited. Third, we did not have the detailed medical records and laboratory data for each patient. We could not evaluate the correlation of cholesterol level and major bleeding risk. Fourth, the indication of each medication might result in non-random treatment allocation. Although the inverse probability of treatment weighting was used in our study to account for this bias, confounding could still exist. Last but not least, DOAC dosage was beyond the scope of this study and was not considered in the calculations.

## Conclusion

Compared with DOACs use alone, concurrent statins and DOACs use decreased the risk of major bleeding, including ICH and GI bleeding. High-intensity statins tended to be more beneficial than low-to-moderate-intensity statins in decreasing the incidence of ICH, especially among females. Statins should be considered in all eligible patients with non-valvular AF who are taking DOACs.

## Data availability statement

The data analyzed in this study is subject to the following licenses/restrictions: The data repository of NHIRD is Health and Welfare Data Science Center, Taiwan. Researchers can obtain the data through formal application to the Health and Welfare Data Science Center, Department of Statistics, Ministry of Health and Welfare, Taiwan (https://dep.mohw.gov.tw/DOS/np-2500-113.html). Requests to access these datasets should be directed to https://dep.mohw.gov.tw/DOS/np-2500-113.html.

## Ethics statement

The studies involving human participants were reviewed and approved by Institutional review board of the Chang Gung Memorial Hospital, Taoyuan, Taiwan. Written informed consent for participation was not required for this study in accordance with the national legislation and the institutional requirements.

## Author contributions

H-HW and T-YC: study design and manuscript writing. S-HC, C-HL, and H-TT: data acquisition. H-HW and H-TT: data analysis. T-HL: research mentorship. Each author contributed important intellectual content during manuscript drafting or revision and accepts accountability for the overall work by ensuring that questions pertaining to the accuracy or integrity of any portion of the work are appropriately investigated and resolved.

## Funding

This work was supported through a grant, CMRPG5D0091, from the Chang Gung Medical Research Council. The funders had no role in study design or interpretation of the findings.

## Conflict of interest

The authors declare that the research was conducted in the absence of any commercial or financial relationships that could be construed as a potential conflict of interest.

## Publisher's note

All claims expressed in this article are solely those of the authors and do not necessarily represent those of their affiliated organizations, or those of the publisher, the editors and the reviewers. Any product that may be evaluated in this article, or claim that may be made by its manufacturer, is not guaranteed or endorsed by the publisher.
