# Supplementary material for: Concurrent use of statins decreases major bleeding and intracerebral hemorrhage in non-valvular atrial fibrillation patients taking direct oral anticoagulants—A nationwide cohort study
Source: Front Cardiovasc Med. 2022 Aug 8;9:969259. doi: 10.3389/fcvm.2022.969259 (PMC9393418; doi:10.3389/fcvm.2022.969259)
Supplement: Supplementary file 1 [file Data_Sheet_1.PDF]

Table S1. Statin Classification According to Intensity

Low-to-moderate-intensity Statins	High-intensity Statins
Fluvastatin 20 to 80 mg	Atorvastatin 40 to 80 mg
Lovastatin 20 to 40 mg	Rosuvastatin 20 to 40 mg
Atorvastatin 10 to 20 mg	
Pitavastatin 1 to 4 mg	
Pravastatin 10 to 80 mg	
Rosuvastatin 5 to 10 mg	
Simvastatin 10 to 40 mg	

Table S2. Risk Scoring System for Stroke and Bleeding

**Risk Scoring system**	**Point if present**
**CHA_2DS_2-VASc score**	
Congestive heart failure	1
Hypertension	1
Age≧65	1
Age≧75	2
Diabetes mellitus	1
Prior stroke or transient ischemic attack	2
Vascular disease*	1
Female	1
**HAS-BLED score**	
Hypertension	1
Chronic kidney disease	1
Liver disease	1
Stroke	1
History of bleeding	1
Age>65	1
Antiplatelet drug using	1
Non-steroidal anti-inflammation drug using	1
Labile INR (not applicable in our study)	1

*include: myocardial infarction, peripheral vascular disease, aortic plaque, receiving percutaneous coronary intervention or coronary artery bypass surgery; INR: international normalized ratio

Table S3. Definitions and codes of comorbidities and outcomes

	**Enhanced ICD-9-CM**	**ICD-10**	Order code
Atrial fibrillation	427.31	I48.0, I48.2, I48.91	
Hypertension	401, 402	I10-I16	
Myocardial infarction	410.x, 412.x	I21.x, I22.x, I25.2	
Congestive heart failure	398.91, 402.01, 402.11, 402.91,404.01, 404.03, 404.11, 404.13, 404.91, 404.93, 425.4–425.9, 428.x	I09.9, I11.0, I13.0, I13.2, I25.5, I42.0, I42.5–I42.9, I43.x, I50.x, P29.0	
Aortic plaque	440	I70	
Percutaneous coronary intervention			33076A, 33076B, 33077A, 33077B, 33078A, 33078B
Coronary artery bypass surgery			68023A, 68023B, 68024A, 68024B, 68025A, 68025B
Peripheral vascular disease	093.0, 437.3, 440.x, 441.x, 443.1–443.9, 47.1, 557.1, 557.9, V43.4	I70.x, I71.x, I73.1, I73.8, I73.9, I77.1, I79.0, I79.2, K55.1, K55.8, K55.9, Z95.8, Z95.9	
Cerebrovascular disease	362.34, 430.x–438.x	G45.x, G46.x, H34.0, I60.x–I69.x	
Ischemic stroke	433-434, 436, 852,853	I67.89, I63-I64, G458-459, S01.90XA, S06.4X0A-S06.4X9A, S06.5X0A-S06.5X9A, S06.6X0A-	

		S06.6X9A, S06.340A-S06.349A, S06.350A-S06.359A, S06.360A-S06.369A	
Transient ischemic attack	435	G45	
Hemiplegia or paraplegia	334.1, 342.x, 343.x, 344.0– 344.6, 344.9	G04.1, G11.4, G80.1, G80.2, G81.x, G82.x, G83.0–G83.4, G83.9	
Dementia	290.x, 294.1, 331.2	F00.x–F03.x, F05.1, G30.x, G31.1	
Diabetes mellitus (DM)	250	E10.0;E10.1;E10.9;E11.0;E11.1; E11.9	
Diabetes with complications	250.4–250.7	E10.2–E10.5, E10.7, E11.2–E11.5, E11.7, E12.2–E12.5, E12.7, E13.2–E13.5, E13.7, E14.2–E14.5, E14.7	
Chronic pulmonary disease	416.8, 416.9, 490.x–505.x, 506.4, 508.1, 508.8	I27.8, I27.9, J40.x–J47.x, J60.x–J67.x, J68.4, J70.1, J70.3	
Chronic obstructive pulmonary disease	490,491.0,491.1,491.2,491.20, 491.21, 491.22, 491.8, 491.9, 492.0, 492.8, 494, 494.0, 494.1, 496	J40, J41.0, J41.1, J41.8, J42, J43.0, J43.1, J43.2, J43.8, J43.9, J44.0, J44.1, J44.9, J47.0, J47.1, J47.9	
Peptic ulcer disease	531.x–534.x	K25.x–K28.x	
Mild liver disease	070.22, 070.23, 070.32, 070.33, 070.44, 070.54, 070.6, 070.9, 570.x,571.x,573.3,573.4,573.8,	B18.x, K70.0–K70.3, K70.9, K71.3–K71.5,K71.7,K73.x,K74.x,K76.0, K76.2–K76.4,K76.8,K76.9,Z94.4	

	573.9		
Moderate or severe liver disease	456.0–456.2, 572.2–572.8	I85.0, I85.9, I86.4, I98.2, K70.4, K71.1, K72.1, K72.9, K76.5, K76.6, K76.7	
Any malignancy, including lymphoma and leukemia, except malignant neoplasm of skin	140.x–172.x, 174.x–195.8, 200.x–208.x, 238.6	C00.x–C26.x, C30.x–C34.x, C37.x–C41.x, C43.x, C45.x–C58.x, C60.x–C76.x, C81.x–C85.x, C88.x, C90.x– C97.x	
Metastatic solid tumor	196.x–199.x	C77.x–C80.x	
Human immunodeficiencyvirus infection	042.x–044.x	B20.x–B22.x, B24.x	
Anemia	280-285	D46.1, D46.4, D50-D64	
Rheumatic disease	446.5, 710.0–710.4, 714.0– 714.2, 714.8, 725.x	M05.x, M06.x, M31.5, M32.x–M34.x, M35.1, M35.3, M36.0	
Chronic kidney disease	580,581,582,583,584,585,586, 587, 588, 589	I12, I13,N00-N05,N07,N11,N14, N17, N18, N19, Q61	
Bone Fractures due to traffic accidents	800-829, E800-E848		
**Major bleeding**			
Intracranial	430, 431, 432.0, 432.1, 432.9, 852.0, 852.2, 852.4, 853.0	I60, I61, I62, S06.340A-S06.349A, S06.350A-S06.359A, S06.360A-06.369A, S06.4X0A-S06.4X9A, S06.5X0A-S06.5X9A, S06.6X0A-S06.6X9A	

Gastrointestinal	530.7, 531, 531.2, 531.4, 531.6, 532, 532.2, 532.4, 532.6, 533, 533.2, 533.4, 533.6, 534, 534.2, 534.4, 534.6, 569.3, 535.01, 535.11, 535.21, 535.31, 535.41, 535.51, 535.61, 535.71, 537.83, 537.84, 562.02, 562.03, 562.12, 562.13, 569.85, 578	K22.6, K25-K28, K29.01, K29.21, K29.31, K29.41, K29.51, K29.61, K29.71,K29.81,K29.91,K31.811, K31.82, K52.81, K55.21, K56.60, K57.01, K57.11, K57.13, K57.21, K57.31, K57.33, K57.81, K57.91,K57.93,K62.5,K92.0,K92.1, K92.2	
Genitourinary tract bleeding	599.7, 626.8	R31.9, N93.8, N93.8	
Other sites	336.1, 363.6, 372.72, 376.32, 377.42, 379.23 (eye); 459, 593.81, 719.1 (joint); 729.92 (soft tissue); 423.0 (heart), 772.5 (adrenal gland)	G95.11, G95.19, H05.23, H11.3, H31.3,H43.1,H47.02,I31.2,M25.0 R58 S37.012A, S37.019A, S37.021A, S37.022A, S37.029A, S37.031A, S37.032A, S37.039A, S37.041A, S37.042A, S37.049A, S37.051A, S37.052A, S37.059A	

Supplementary Figure

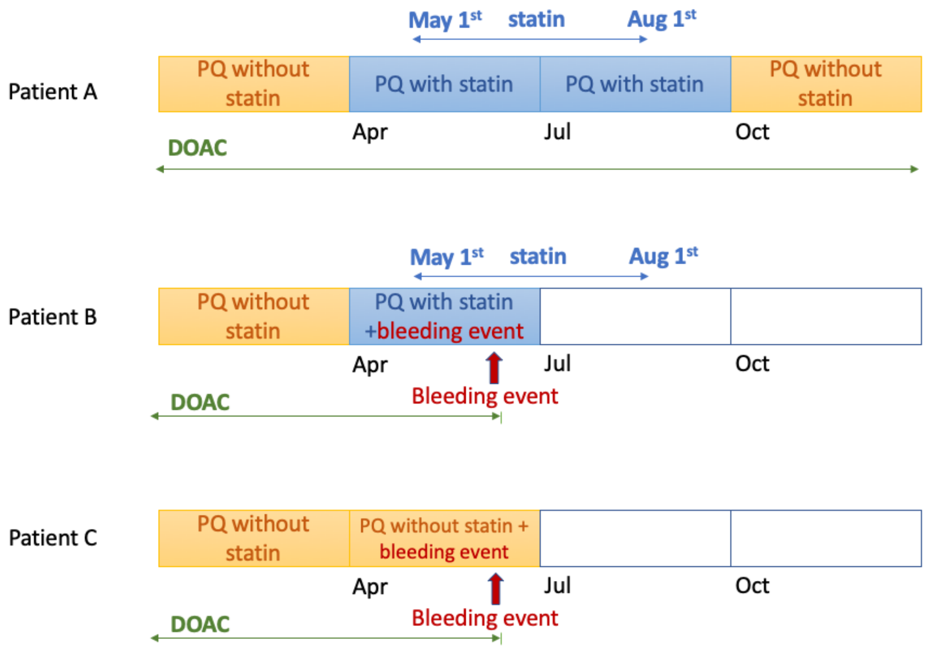

Supplement Figure. Illustration of Person-quarter Examples.

Examples of person-quarters (PQs) counting. In this year, patient A had two person-quarters of direct oral anticoagulant (DOAC) exposure without concurrent statin use, and two person-quarters of DOAC and statin exposure; patient B has one person-quarter of DOAC without concurrent statin and one person-quarter of DOAC with concurrent statin and bleeding event; patient C has two person-quarters of DOAC without concurrent statin; bleeding event occurred in one person-quarter. Within the PQ, the time-points of DOAC prescription, statin prescription, and bleeding events were all recorded, as to ensure that only bleeding events after DOAC prescription were counted, and only bleedings after statin prescription were recorded as bleeding events with statin using.
